# Treatment of Mandibular Fractures by Two Perpendicular Mini-Plates

**Published:** 2014-01

**Authors:** Amin Rahpeyma, Saeedeh Khajehahmadi, Sadegh Barkhori Mehni

**Affiliations:** 1 *Oral and Maxillofacial Diseases Research Center, School of Dentistry, Mashhad University of Medical Sciences, Mashhad, Iran.*; 2 *Dental Research Center, School of Dentistry, Mashhad University of Medical Sciences, Mashhad, Iran.*

**Keywords:** Jaw Fixation Techniques, Mandibular Fracture, Mini-plate

## Abstract

**Introduction:**

In open reduction and internal fixation for the treatment of mandibular fracture, the fixation technique used is very important in reducing post-operative complications and promoting the healing process. This study assessed the results of fixation of the mandible using two mini-plates perpendicular to each other in the lower border of the mandible for fracture treatment.

**Materials and Methods:**

Access to the fractures was via an extraoral approach (through existing scars or incisions). After reductions of mandibular fractures, the fracture line fixation was accomplished using two mini-plates perpendicular to each other. One-week intermaxillary fixation (IMF) was applied and 3 weeks of soft diet was recommended in the post-operative period. All patients were followed up for at least 1 year regarding infection and malocclusion.

**Results:**

Twenty-five patients (28 fracture lines) underwent this technique. Most (81.8%) patients were male and the mean age was 41.3±7.59 years (range, 17–73 years). Symphyseal fracture (frequency, 52%) was the most prevalent followed by angle (32%) and body (16%) fractures. Among the patients who underwent surgery, only one malocclusion and no cases of infection were observed. No cases of facial nerve weakness or damage were observed in this study.

**Conclusion:**

***This method can be used in specific cases to replace treatment with one mini-plate, which necessitates a more intensive fixation or reconstruction plate therapy. ***

## Introduction

After zygomaticomaxillary complex fracture, the mandible fracture is the second most frequent maxillofacial injury associated with all-terrain vehicle (ATV) collisions (1). The treatment of a mandible fracture depends on several factors including the extent of displacement, soft and hard tissue loss, tooth conditions, and the capabilities of the responsible doctors and hospital facilities (2). Treatment of a mandible fracture is complex for a number of reasons. The involvement of muscles, especially masticating muscles, which affects the re-positioning of the fracture parts after a fracture, involvement of teeth and inferior alveolar nerve, and the dental status (including temporary, permanent, and mixed teeth, and complete or partial teeth loss) makes the treatment of fracture of mandible difficult and challenging (2,3). As one of the aims of treatment is restoration of occlusion, determination of the position of the occlusion before fracture and the presence of malocclusion [class I, II, III (deep or open bite)] is extremely important in devising a suitable surgical strategy (4). These abnormalities can be diagnosed by enquiring about the overlay of the teeth before the fracture, examination of dental systems, radiography, and pre-trauma photography. While an observational approach is the most suitable for greenstick fractures without occlusion impairment, fractures without or with minimal displacement are better treated by closed treatment. However, occlusion is commonly restored by wiring, the Ivy system, an arch bar, or intermaxillary fixation (IMF) (5). Open strategies with or without internal and external fixation are the two other treatment modalities applied for fracture of the mandible (6,7). In more complicated cases such as multiple fractures accompanying condylar fracture, nerve entrapment, great displacement, co-fractures and poor compliance, internal fixation is recommended. In these situations, after exposure of the fracture line, the parts are overlaid. Next, alignment is maintained by wire and screw (titanium and bio-absorbable). Application of wires necessitates a longer fixation time in comparison with titanium plates, as the second approach efficiently endures mastication pressure until complete healing of the bone (8). Plates used for the treatment of a mandible fracture include micro-plates, mini-plates (1.3-,2-mm thickness), locking mini-plates, reconstru- ction, fracture plates, THORP system, and the compression system(9,10).

Application of internal fixation tools, especially plates, in the treatment of a mandibular fracture conforms to special biomechanics rules and simply overlaying fracture parts beside each other is not sufficient (11). The first plating system in maxillofacial surgery was introduced in 1973 by Champy and Michelet (12), who placed plates in the tension area of the mandible angle and symphysis to prevent displacement of the fracture parts of the upper border. Plates were inserted in the lower border in the premolar region which was assumed to be the compression area if the patient had teeth. Otherwise, damage to teeth was inevitable. However, following a better comprehension of the tension and compression areas, defined according to the place of energy insertion, it is now possible to insert the plates into the lower border of mandibular fracture (11). Awareness of the distance between the inferior alveolar nerve and the lower border is necessary to prevent damage to the nerve and any subsequent legal issues. In a study by Rajchel et al, (13) the lowest distance was documented in the first molar and second premolar teeth (7.5±1.5mm). In this investigation, we assessed an apparently more cost-effective open surgical treatment approach for fractures of mandible. 

## Materials and Methods

This study was designed to evaluate a treatment method involving application of two perpendicular mini-plates. Twenty-five patients with a mandible fracture (symphysis, body or angle fracture of mandible, one- or two-sided, with or without condylar fractures) referred to Shahid Kamyab Hospital, Mashhad University of Medical Sciences, Mashhad, Iran from July 2006 to September 2010 were selected to be treated by open reduction with internal fixation using two perpendicular mini-plates. All procedures were approved by the institutional ethical committee and informed consent was obtained from all patients. One mini-plate was placed in the lower border and the other was positioned perpendicular to it on the lateral surface of the bone. The inclusion criteria were symphysis, body or angle fracture of mandible, either one-sided or two-sided. Exclusion criteria were presence of comminuted fracture, mixed dentition, mid-face fracture, and any systemic disease. 

**Fig 1:**
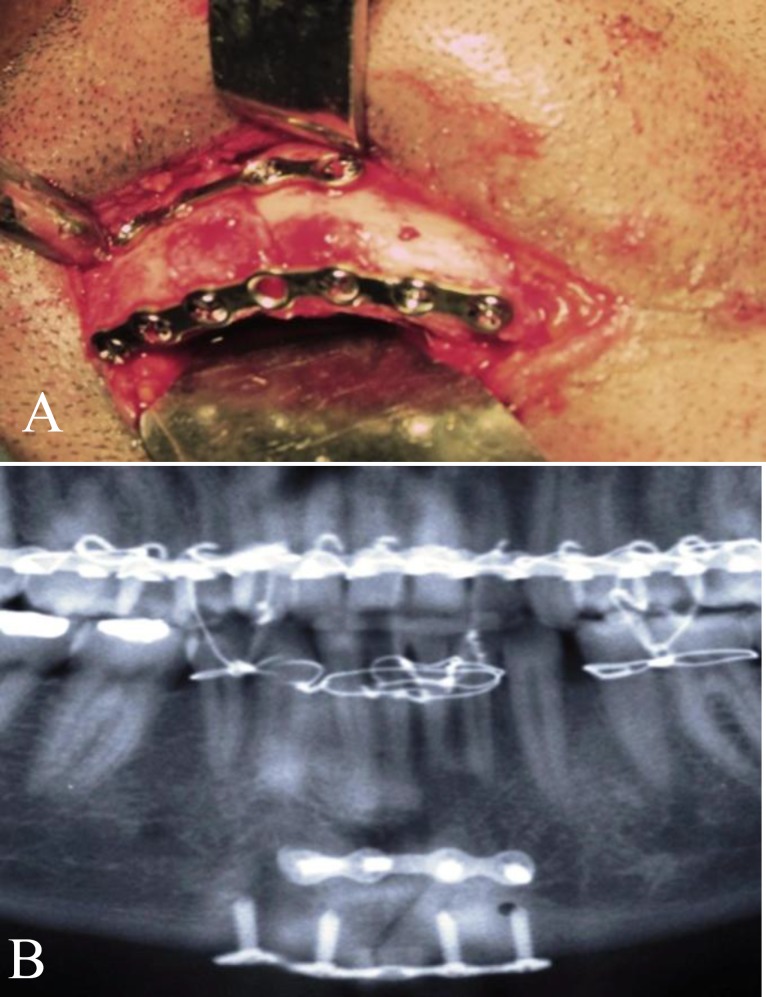
a) Clinical picture of two perpendicular mini-plates in mandibular symphyseal fracture fixation; b) Orthopantomograph of another patient with symphyseal fracture treated by two perpendicular mini-plates.

After general anesthesia, the existing scar or extra oral incision (sub-mental, sub-mandibular, Risdon) was used for access to the fracture lines depending on the place of fracture. Upper and lower jaws were fixed by upper and lower jaw arch bars or other dental wiring techniques. Then we injected Lidocaine (E 1/80000) and dissected the skin and subcutaneous tissue layer by layer with careful attention to save the facial nerve branches. If necessary, the facial artery or vein was ligated. When we had accessed the bone, occlusion and inter-dental fixation were performed to render osteosynthesis and internal fixation with the two mini-plates (using two or three screws, each beside the fracture line). The mini-plate that was placed in the lower border from below (first plate) had four or six holes and a bar thickness of 2 mm. In the symphyseal area we used a six-hole orbital plate. The curve of the orbital mini-plate was easily adapted with the lower border mandibular curve in the symphyseal area. Two or three mini screws were placed in each side of the fracture line. At up to 10 mm, the screw length in the mandibular symphysis could be long. In body and angle fracture fixation these screws should be shorter in order to avoid the inferior alveolar nerve and damage to the artery. Depending on the distance between the outer lower mandibular cortex and the lower part of the inferior dental canal in the orthopanto- mogram (OPG), we selected the appropriate screw length. Increasing the screw length was not necessary because its tip was embedded in cancellous bone and an increase in length did not lead to bicortical fixation. We recommend a length of 6 mm in the mandibular body and angle area. The second plate was placed on the lateral surface of the lower border, while the whole width of the lower border was considered for screw length selection. Especial attention was paid to avoiding screw collision. After internal fixation and full irrigation of the site, four layers (periosteum, muscle, subcutaneous, and skin) were sutured (Fig. 1a).

All patients experienced a 1-week maxillomandibular fixation. Patients’ post-operative diet was restricted to liquids in the first week and a soft diet for one month. Arch bar and other dental wiring techniques including Ivy loop and Essig wirings were removed 6 weeks after the operation. Patients were followed clinically regarding occlusion and infection after 1 week, 2 weeks, 1 month, 6 months, and 1 year. The site of surgery was examined by direct observation by the clinician. Radiological evaluation was performed after surgery and 6 months later to assess the healing process. 

## Results

Most patients (81.8%) were male and the mean age of the sample was 41.3 years (range, 17–73 years). One-sided symphysis fractures, two-sided symphysis fractures, body, and angle fractures were found in 10 (40%), three (12%), four (16%), and eight (32%) patients, respectively. Eight cases had accompanying mandibular fractures in the condyles; five of which were one-sided. Access to the fracture line was through lacerations caused by trauma in nine patients (36%). In other patients sub-mental, sub-mandibular, and Ridson approaches were selected for symphysis, body, and angle of mandible, respectively; in three edentulous patients this technique used but was not included in this study. 

All 25 patients treated using this method were followed for 1 year. Restoration of appropriate occlusion was achieved in most patients (96%), and only one case acquired malocclusion. This case was a 52-year old man with a two-sided symphysis fracture and a left side condylar co-fracture. Post-surgical infection was not observed in our patients. 

## Discussion

The surgical technique applied in this survey, using two perpendicular mini-plates for treatment of fracture of the mandible, seems to be acceptable as no cases of post-surgical infection and minimal malocclusion were found. It seems that this method can be assumed to be an appropriate alternative to treatment with reconstruction plates because it does not require heavy fixation and may be more cost-effective. 

This study shows that risk of infection with this technique is minimal. Infection after mini-plate osteosynthesis for mandibular fractures was 1% in another study by Nakamura et al (14). However, it should be noticed that oral hygiene, connection between fracture line and teeth, antibiotic therapy, and the fixation technique contribute to the incidence of infection (3). Nowadays, as antibiotics are prescribed according to the available guidelines, it seems that the fixation technique, which greatly affects stabilization of fracture parts and efficacy of recruitment of a blood supply and healing process, plays the most important role in the development of post-surgical infection. The risk of infection was 7.5% by reconstruction plates, 32% by dynamic compression plates, and 25% by mandibular mini-plates in mandibular angle fracture (10,15,16) .When compared with other internal fixation techniques, it can be seen that infection is not a problem with this method. The low risk of infection associated with this technique is attributed to the exclusion of patients affected by systemic diseases, comminute fractures, and the avoidance of intraoral incisions. 

The low rate of malocclusion associated with this method (<5%) is satisfactory when compared with the results of other techniques. In the study by Nakamura, the rate of malocclusion after insertion of mini-plates was nearly 4% (14). The confounding role of condylar co-fractures should also be considered (17) .There is evidence to support application of two perpendicular mini-plates for the treatment of a mandibular fracture, especially a condylar fracture. In 2002, Wagner studied the biomechanics of forces in the condylar fracture and concluded that open surgery and internal fixation by two perpendicular mini-plates should be recommended (18). It is accepted that tension-compression zones in a fractured mandible will change as the applied force is exerted posterior to the fracture line or muscular axis in comparison with its application anterior to the fracture line (19). This observation coupled with the fact that patients with a fractured mandible do not apply normal bite forces for several weeks to months (20) support the basic premise of the use of two perpendicular mini-plates in the treatment of mandibular fractures with emphasis on the rigid inferior border plating without compression with low profile plates and three-dimensional fixation.

However, there are some considerations to be taken into account with this method. Because there is a possibility of damage to the alveolar nerve, the distance between the nerve and the upper border of the bone is of significance in several studies (21,22); however, in our study the distance to the lower border was much more important. The reason for this is that the second plate, perpendicular to the first, as well as the screws, is inserted into the lower border. Thus, awareness of the distance between the lower alveolar nerve and the lower border is necessary to prevent damage to the nerve, lower lip anesthesia, and any subsequent legal issue. Symphyseal fracture in our group was more prevalent (unilateral and bilateral, 52%); sub-mental laceration encourages extraoral approaches. Ordinary intraoral incisions are used for access to the fracture line of mandible in order to avoid a visible scar and facial nerve injury. Our technique requires an extraoral approach and is used when displacement of the fracture fragments are such that intraoral approaches cannot properly reduce the fracture. Direct visualization of the fracture line with buccal and lingual fracture alignment explains the low percentage of malocclusion that is seen in this study (4%).

The two perpendicular mini-plate techni- ques can three-dimensionally control the fracture lines. This technique has a low cost, low stress shielding, and low plate profile.

Lower lip sensory changes after internal fixation of mandibular fractures is a concern to surgeons. In post-operative OPGs, there was no case of inferior dental canal penetration by first plate screws (Fig. 1b). Lip sensation 6 months after the procedure was normal in 22 patients, while three patients (two angle and one body fracture) had paresthesia of the lower lip on the fracture side (12%). This can be attributed to displacement of bony fragments after trauma and before surgery, and is not related to this technique. 

## Conclusion

Insertion of two perpendicular mini-plates for fracture therapy of the mandible is an efficient procedure that has only minimal complications, although it seems other studies need to be performed for this method to be accepted as a replacement for current treatment approaches. The technique may also be used for mandibulotomy osteosy- nthesis in the treatment of pathologic lesions located medial to the mandible, and is especially useful in edentulous fracture fixation in which the low profile of the plates and inferior border placement of the mini-plates prevents their removal for prosthodontic purposes. This method can be used instead of a single mini-plate or reconstruction plate in clinical situations that need both intensive fixation and rigidity. 
